# Research on Obstacle-Crossing Performance of a Passive Rocker-Bogie Six-Wheel Mobile Platform for Nuclear Environments: Analysis Based on Onboard Sensors

**DOI:** 10.3390/s26144558

**Published:** 2026-07-18

**Authors:** Jun Liu, Qian Deng, Shihua Liu, Shuntao He, Shuliang Zou

**Affiliations:** 1 College of Mechanical Engineering, University of South China, Hengyang 421001, China; yl11057@163.com (J.L.);; 2 Hunan Provincial Key Laboratory of Emergency Safety Technology and Equipment for Nuclear Facilities, University of South China, Hengyang 421001, China

**Keywords:** nuclear decommissioning, rocker-bogie suspension, six-wheel mobile robot, onboard torque sensing, multibody dynamics

## Abstract

To address the inefficiency of demolition robots at nuclear contamination sites due to frequent retreats to safe zones for attachment replacement, this study develops and experimentally evaluates a six-wheeled mobile platform for attachment-replacement support near the work area. Structurally, the prototype adopts a well-established passive rocker-bogie suspension architecture combined with six-wheel independent drive. The focus of this work is not to claim a new suspension topology, but to evaluate its engineering feasibility and drive-load margins for a heavy-duty nuclear support platform through multibody simulation and onboard-sensor measurements. A constrained multibody model was implemented in ADAMS/Simulink to represent rocker joints, wheel revolute joints, actuator limits, and wheel–ground contact. A full-scale prototype was tested on representative nuclear-facility terrain conditions, including a 20° slope and a 250 mm vertical step. The results show that the prototype completed both tests while the measured motor torques remained within the allowable drive range. The positive and negative torque signs observed on the left and right sides are explained by mirrored motor installation and coordinate definitions rather than by a special torque-distribution mechanism. This study provides a structural selection and experimental performance reference for mobile operation support in radiation environments.

## 1. Introduction

Mobile robots are playing an increasingly critical role in high-risk environments such as nuclear facility decommissioning, post-disaster rescue, and space exploration [1]. These systems not only replace humans in executing complex tasks within radioactive or toxic zones but also significantly enhance operational efficiency and precision [2,3,4]. This is particularly evident in nuclear Decommissioning and Dismantling missions, where environments are highly unstructured, featuring scattered debris, ruins, and various obstacles. Furthermore, robots are often required to carry heavy-duty payloads such as hydraulic shears and grapples and perform tool switching between different operational stages. However, current operational modes rely heavily on manual or semi-automatic control. Limited by the mobility performance of standard chassis, robots often struggle to maintain a stable posture for operations or tool changing on rugged, in situ terrain. Consequently, operators are forced to frequently intervene or move the equipment to flat “safe zones” for retooling. This necessity not only reduces efficiency but also increases the risk of radiation exposure for personnel [5,6,7].

Therefore, developing a mobile platform capable of adapting to complex unstructured terrains while maintaining high load capacity and agility is a fundamental prerequisite for enhancing nuclear robotic capabilities. Traditional tracked chassis, while offering strong traction, suffer from poor maneuverability in confined spaces and can damage ground surfaces. Conversely, conventional differential wheeled or rigid suspension chassis, despite their simplicity, struggle to compensate for significant terrain variations—such as steps or steep slopes—through structural compliance. This often leads to wheel lift-off, slippage, or tipping, failing to meet the stability requirements for high-precision operations [8,9,10,11]. In contrast, a six-wheeled mobile platform integrating independent driving with a passive rocker-bogie suspension mechanism [12,13,14,15] is a practical candidate configuration because of its terrain adaptation and load-bearing potential. Recent studies have further examined standard and modified rocker-type suspensions for obstacle negotiation. Ugenti et al. compared elastic and inelastic rocker-type suspensions and showed that adding compliance to a rocker mechanism can reduce the friction and drive torque requirements during obstacle climbing, highlighting the continuing relevance of rocker-based passive suspension design for rough-terrain robots [16].

In recent years, extensive research has been conducted on the mechanism design, dynamic control, and obstacle-crossing stability of multi-wheeled mobile robots. Regarding mechanical configuration and mobility verification, Arnold et al. [17] proposed the high-load robot LEVA, integrating an advanced leg-type suspension system to validate its off-road transport capabilities. Similarly, to enhance chassis stability, Shangfei L et al. [18,19] designed a multifunctional six-wheeled rescue robot featuring an air-balanced single-leg structure and multimodal control. Expanding on environmental adaptability, Zhao J et al. [20] investigated a wheeled-legged hybrid robot (WLHR), confirming its motion performance through ADAMS kinematic simulations and field experiments on variable terrains.

Focusing on suspension system optimization and obstacle negotiation mechanisms, Zhe S et al. [21] analyzed a passive six-wheeled robot equipped with rocker arms and established a dynamic model for step traversal. To further improve trafficability, Sanfeng H et al. [22] introduced a novel active–passive suspension configuration, validating its vertical obstacle-crossing performance. In the context of planetary exploration, He J et al. [23] proposed an active–passive coupled leg mechanism, validating the control strategy through simulations. Addressing extreme conditions such as subsidence, Baofeng Y et al. [24] designed an active suspension system for Mars rovers, developing specific control strategies for creep escape and wheel lift escape.

In the domain of dynamic modeling and mathematical optimization, researchers have integrated terramechanics to enhance control precision. For rough-terrain robots with passive articulated suspension, Grazioso et al. developed and validated a multibody model of a tracked robot in MSC Adams, demonstrating that a validated multibody digital model can be used to evaluate suspension configurations and predict field performance before prototype optimization [25]. Yingbiao W et al. [26] conducted a comprehensive structural stability and kinematic analysis of a six-wheel rocker robot. Incorporating classical ground contact mechanics, Dawei G et al. [27] established a dynamic model that accounts for slip and sliding terms. To address whole-body motion under rolling constraints, Kameduła M et al. [28] introduced a first-order inverse kinematics scheme and a damping method for singularity management. Furthermore, Junqiang Z et al. [29] developed a mechanical model for wheel–ground interaction in a wheel–step composite mobility system.

Numerical optimization tools have also been widely applied to structural design. Fengchen W et al. [30] utilized ADAMS software to perform multi-objective optimization on key dimensions of an all-terrain robot. Noble S et al. [31] proposed an enhanced optimization formula specifically for rocker-bogie suspension configurations. Finally, Chaoxing W et al. [32] developed a detailed dynamic model to analyze performance boundaries during the obstacle-crossing process.

Although recent studies have advanced the analysis of rocker-type suspension obstacle negotiation and multibody modeling of passive articulated mobile robots, most of them focus on planetary/agricultural rough-terrain robots, compliant rocker mechanisms, or tracked platforms. Their findings provide valuable modeling and design references, but they do not directly address a heavy-duty six-wheel passive rocker-bogie platform for nuclear decommissioning support, especially one validated through onboard motor torque sensing under slope climbing and vertical step traversal conditions.

To address these challenges, this paper designs and tests a six-wheeled mobile platform tailored for complex nuclear environments and based on a passive rocker-bogie suspension system. Rather than presenting the suspension architecture as a new topology, the study focuses on its adaptation to a heavy-duty nuclear support scenario and on the consistency between multibody simulation and onboard torque measurements. A constrained multibody simulation model is established to represent the rocker joints, wheel-drive joints, actuator limits, and wheel–ground contact during slope climbing and vertical obstacle negotiation. A full-scale physical prototype is then tested to evaluate trafficability and drive-load margins under representative terrain conditions.

The main contributions of this paper are summarized as follows:

A constrained multibody simulation framework for obstacle negotiation is established. The model represents the passive rocker-bogie mechanism through joint constraints and contact interactions, and it is used to estimate wheel torque demand during slope climbing and vertical step traversal.

Experimental validation of drive torque response under representative terrain conditions is established. Based on a full-scale engineering prototype, this study records onboard motor-current-derived torque during 20° slope climbing and 250 mm step traversal. The left–right sign difference in the measured torque is identified as a consequence of mirrored motor installation and coordinate definition, while the magnitudes are used to assess drive-load margins.

Validation of system engineering feasibility for heavy-duty nuclear applications is established. The experiments show that the prototype can complete the specified slope and step tests without prolonged motor overload. Because direct wheel-normal-force measurements were not included in the present prototype, this study does not claim measured equal load distribution among all six wheels; wheel–ground contact is inferred only indirectly from torque continuity and observed motion.

## 2. Mechatronic System Design

### 2.1. Design Requirements and Overview

#### 2.1.1. Application Scenario Analysis

This study targets high-risk unstructured environments such as nuclear facility decommissioning and post-disaster rescue. These environments are characterized by:

1. Confined spaces and sharp turns: narrow equipment corridors require the robot to possess high maneuverability, specifically the ability to perform zero-radius turns.
2. Complex obstacles: the ground is scattered with construction debris, residual piping, and industrial steps, demanding superior obstacle-crossing performance.
3. Discontinuous surfaces: the presence of trenches or protrusions requires the chassis to reduce wheel lift-off and help preserve traction continuity.

Addressing these challenges, traditional four-wheeled chassis are prone to wheel lift-off and slippage, while tracked robots suffer from excessive weight, high energy consumption, and maintenance difficulties. Therefore, this study proposes a high-maneuverability mobile platform based on a “Six-Wheel Independent Drive (6WD) + Passive Rocker Suspension” configuration.

#### 2.1.2. Key Design Specifications

Based on practical engineering requirements, the core performance specifications are defined.

1. Obstacle height: ≥250 mm (vertical step).
2. Climbing capability: ≥20° (concrete surface).
3. Steering mode: differential steering, turning radius R_turn_ = 0.
4. Payload capacity: ≥180 kg.

### 2.2. Mechanical Structure Design

**Figure 1:**
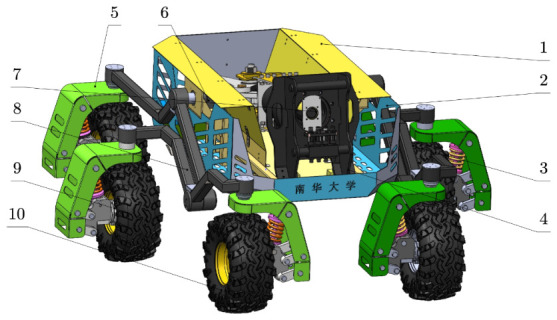
Overall structural diagram: 1. cargo platform; 2. vehicle body; 3. rear swing arm; 4. chassis structure; 5. independent suspension; 6. rotary motor; 7. working attachments; 8. front swing arm; 9. wheel hub motor; 10. drive wheel.

To achieve the aforementioned specifications, the mechanical system adopts a modular design, primarily consisting of the main chassis frame, six independent drive units, and the passive rocker suspension mechanism, as shown in Figure 1.

#### 2.2.1. Locomotion Configuration and Tire Selection

Unlike laterally movable platforms that require high ground flatness, this prototype adopts a 6 × 6 differential-steering configuration.

1. Steering mechanism: steering is achieved by controlling the velocity difference (Δv = v_L_ − v_R_) between the left and right wheel groups. When vL = −vR, the robot performs a pivot turn around its geometric center, greatly enhancing agility in confined nuclear environments.
2. Tire selection: wide-section off-road pneumatic tires with a radius of 200 mm were selected.

**Figure 2:**
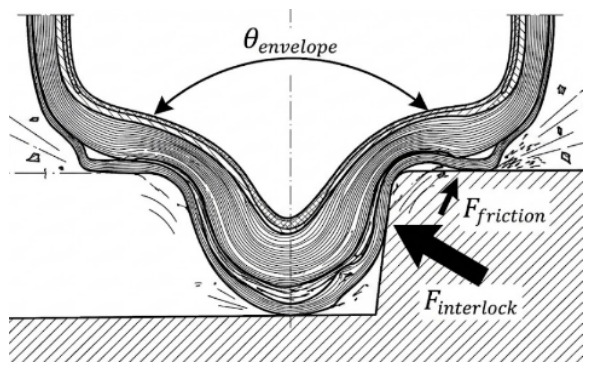
Tire deformation force diagram.

Compared to rigid wheels, pneumatic tires possess significant flexible deformation capabilities. When climbing a 250 mm rigid step, the tire surface can enlarge the contact envelope and improve mechanical interlocking with the step edge, helping reduce slippage, as shown in Figure 2. Additionally, the damping characteristics of the tires help absorb high-frequency vibrations during motion, protecting onboard precision electronics.

#### 2.2.2. Passive Rocker Suspension Mechanism

**Figure 3:**
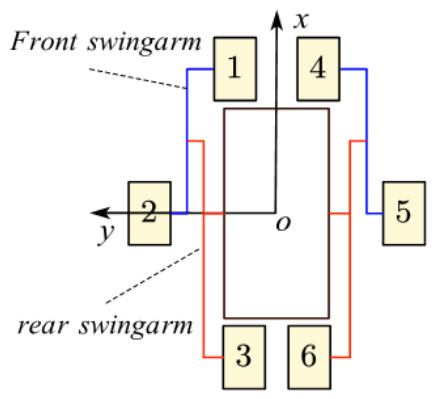
Rocker arm mechanism distribution.

To resolve the issue of “wheel lift-off” common in multi-wheeled robots on uneven terrain, a passive rocker mechanism is configured on each side of the chassis, as shown in Figure 3.

1. Structural layout: the rocker pivot is mounted at the center of the chassis side. The front and middle wheels are mounted on the ends of the rocker, while the rear wheel is fixed to the rear of the chassis.
2. Terrain adaptation principle: the rocker-bogie mechanism uses passive rotation about the rocker pivot to improve terrain conformity. When a wheel encounters a protruding obstacle, the rocker rotates in response to the terrain and helps reduce wheel lift-off and improve contact continuity among the remaining wheels. The present study treats this as a passive mechanical adaptation mechanism; direct wheel-normal-force measurement is not included and is identified as a subject for future work.

### 2.3. Mechatronic and Control System Architecture

**Figure 4:**
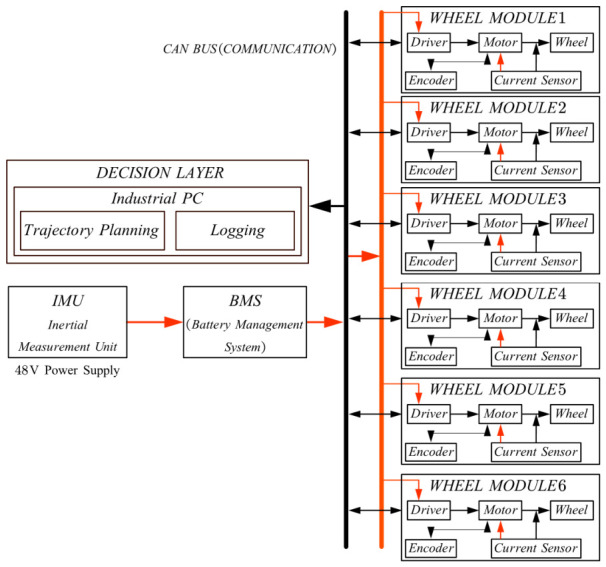
Actuation and sensing layer.

To achieve precise control and state monitoring in nuclear environments characterized by high radiation and electromagnetic interference, the prototype adopts a distributed control architecture based on the CAN bus (Controller Area Network). The system deeply integrates energy management, motion control, and multi-dimensional sensing, as illustrated in Figure 4.

#### 2.3.1. Distributed Hardware Topology

The hardware system primarily consists of an onboard main controller (Industrial PC), six servo drive nodes, and a power management module.

1. Communication bus: the CAN 2.0B industrial CAN bus is selected as the communication backbone of the system. Compared to USB or RS232, the CAN bus utilizes differential signal transmission, providing superior electromagnetic interference immunity, making it highly suitable for reliable data transmission in nuclear environments.
2. Drive nodes: six independent servo drivers are connected in parallel to the CAN bus. Each driver possesses a unique node ID, responsible for receiving speed commands from the controller and performing closed-loop control of the corresponding brushless DC motor.
3. Power system: power is supplied by a 48 V/50 Ah high-rate lithium battery pack. Since differential steering generates instantaneous high-current pulses during pivot turns, the power module integrates a BMS (Battery Management System) to monitor over-current and overheating risks.

#### 2.3.2. Multi-Modal Sensing System

The sensing system is the core for dynamic validation. The platform integrates proprioceptive and environmental sensors to provide real-time feedback on the robot’s motion state and terrain interaction.

1. High-precision joint proprioception:

Position/velocity: an incremental photoelectric encoder (2500 lines) is integrated at the rear of each drive motor. Amplified by the gearbox reduction ratio, the angular resolution at the wheel end is extremely high, enabling the precise capture of minute speed fluctuations (e.g., during slippage).

Torque sensing: the drivers feature built-in high-frequency current sensors (Hall effect) that monitor phase currents at 1 kHz. Via the torque constant Kt, current data are converted into wheel output torque in real time. This is the critical data source for analyzing obstacle-crossing resistance and motor load factors in Section 5.

2. Attitude and inertial sensing: a 9-axis MEMS Inertial Measurement Unit (IMU) is installed at the center of the chassis. It outputs the chassis pitch, roll, and 3-axis acceleration at a frequency of 100 Hz. This is vital for assessing the robot’s stability (e.g., tipping risks) during slope climbing.

#### 2.3.3. Data Flow and Control Strategy

The control system operates on a 50 Hz control cycle. In each cycle, the main controller synchronously sends target speed commands to the six nodes via the CAN bus and simultaneously retrieves data packets containing [Actual Speed, Real-time Current, Encoder Pulses] from each wheel. These data are logged for subsequent comparison with multibody simulation outputs and for evaluating drive-load response under representative terrain conditions.

## 3. Kinematics and Constrained Multibody Dynamics Modeling

This section establishes the kinematic description of the six-wheel mobile platform and the constrained multibody modeling framework used for slope climbing and vertical obstacle negotiation. The model focuses on rocker-joint constraints, wheel-drive joints, and wheel–ground contact rather than on closed-form quasi-static force-balance equations.

### 3.1. Kinematics Analysis

**Figure 5:**
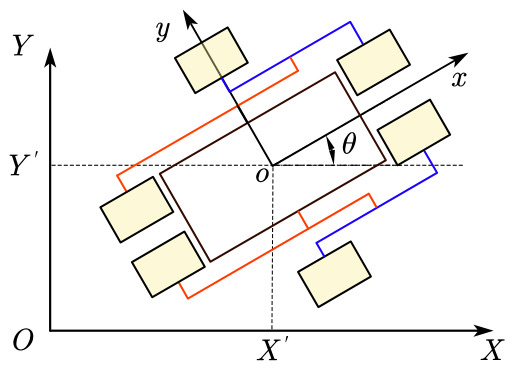
Relative pose between the world coordinate system and the local coordinate system.

The platform is modeled as a differential-steering vehicle. The lateral velocity in the body frame is constrained to zero under pure rolling assumptions. Therefore, the kinematic model only relates the body frame longitudinal velocity and yaw rate to the equivalent left- and right-side wheel-group velocities, as shown in Figure 5.

The motion of the robot body is described by a differential-steering kinematic model. In the local coordinate system xoy, $\dot{x}$ is the longitudinal velocity component of the center point o of the six-wheel differential-steering platform, $\dot{y}$ is constrained to zero under pure rolling assumptions, *θ* denotes the yaw angle, and $\dot{\theta }$ denotes the yaw rate.

In the local coordinate system xoy, the velocity of the six-wheel differential-steering mobile platform is represented by v_b_:

(1) $$\nu_{b}={\left[\begin{array}{c} \dot{x}\\ \dot{y}\\ \dot{\theta } \end{array}\right]}^{T},\;\dot{y}=0$$

The equivalent longitudinal velocities of the left and right wheel groups are calculated from the three independently driven wheels on each side:

(2) $$\left\{\begin{array}{c} v_{L}=\frac{r}{3}\left(\omega_{1}+\omega_{2}+\omega_{3}\right)\\ v_{R}=\frac{r}{3}\left(\omega_{4}+\omega_{5}+\omega_{6}\right) \end{array}\right.$$

**Figure 6:**
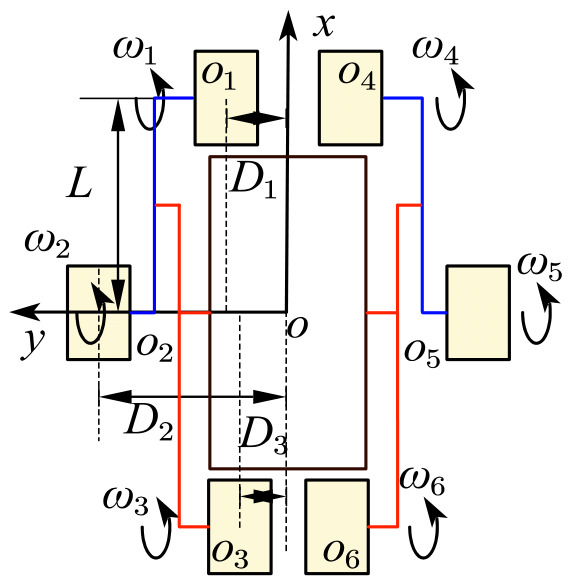
Position parameters of a six-wheel differential-steering mobile platform.

The structure of the chassis on both sides is symmetrical. Based on the geometric parameters defined in Figure 6, the coordinates of the six wheels are expressed relative to the vehicle body center.

For the symmetric six-wheel chassis, the differential-steering relationship between the left/right wheel-group velocities and the platform motion is as follows:

(3) $$\left\{\begin{array}{c} \dot{x}=\frac{1}{2}\left(v_{L}+v_{R}\right)\\ \dot{\theta }=\frac{1}{B}\left(v_{L}-v_{R}\right) \end{array}\right.$$

From the formula, it can be seen that, when the linear velocities of the wheelsets on the left and right sides are equal, the platform moves along a straight line, and the yaw rate is zero; when the velocities of the wheelsets on the left and right sides are equal in magnitude but opposite in direction, the platform rotates around the center of the vehicle body to achieve a turn in place.

### 3.2. Multibody Dynamics Model with Joint Constraints

Obstacle negotiation by a rocker-bogie vehicle is a constrained multibody dynamics problem. Therefore, this study models the vehicle as a set of rigid bodies connected by passive rocker joints and wheel revolute joints, with wheel–ground and wheel–obstacle contact solved in the multibody dynamics environment.

#### 3.2.1. Generalized Coordinates and Constraints

Let q denote the generalized coordinates of the chassis, rocker arms, and six-wheel bodies. The passive rocker pivots and wheel axles are described by holonomic joint constraints Phi(q,t) = 0. These constraints preserve the mechanism geometry while allowing the rocker arms to rotate passively in response to contact forces. The chassis pitch angle, rocker angles, and wheel rotation angles are treated as independent physical variables linked by the joint constraints, rather than being assumed equal to one another.

The constrained equations of motion are written in the standard multibody form M(q) qddot + Phi_q(q,t)^T lambda = Q(q,qdot,t) + Qc(q,qdot,t), with Phi(q,t) = 0. Here, M(q) is the generalized mass matrix, Phi_q is the constraint Jacobian, lambda is the vector of Lagrange multipliers associated with joint reactions, Q contains gravity and motor-drive inputs, and Qc contains contact/friction forces at wheel–ground and wheel–obstacle interfaces.

#### 3.2.2. Contact and Drive Modeling

For each wheel i, contact is activated when the wheel geometry intersects the ground, slope, or step surface. The normal and tangential contact components are solved by the multibody contact model rather than by prescribing internal wheel chassis forces in an external moment-balance equation. The drive torque tau_i is applied at the corresponding wheel revolute joint and is limited by the motor torque constraint used in the ADAMS/Simulink co-simulation.

This formulation avoids treating internal joint reactions between the wheel modules and chassis as external moments. It also avoids equating rocker-link inclination angles with the chassis pitch angle. The quantities compared with experiments in Section 5 are the simulated wheel-drive torques and the measured current-derived motor torques. Direct wheel-normal-force validation was not performed in the present experiment and is therefore not claimed.

#### 3.2.3. Model Outputs Used for Validation

The model outputs used in this paper are the time histories of wheel-drive torque during the 250 mm step traversal and 20° slope climbing scenarios. These outputs are compared with onboard sensor data to evaluate the sequence of wheel loading and the drive-load margin. The multibody simulation is therefore used as a design and validation tool, and no conclusions are drawn from closed-form analytical obstacle-crossing equations.

## 4. Simulation and Experimental Setup

To evaluate the obstacle-crossing and slope climbing performance of the six-wheel mobile platform in typical nuclear environment terrains, this section describes the construction of the constrained multibody simulation environment, the physical prototype, and the experimental scenarios.

### 4.1. Construction of Dynamic Simulation Environment

#### 4.1.1. Model Parameters and Contact Settings

Significant multibody dynamic coupling and strongly nonlinear contact behavior exist during obstacle-crossing and hill climbing: the normal/tangential forces in wheel–ground contact change rapidly with wheel load transfer, attitude changes, and contact state switching, exhibiting obvious time-varying and discontinuous characteristics. This paper adopts a joint simulation approach using ADAMS and Simulink. ADAMS is responsible for high-fidelity multibody dynamics and contact solutions, while Simulink is responsible for control algorithm and actuator constraint modeling. This achieves a closed-loop control mechanism of “speed command–motor output torque–wheel speed feedback,” facilitating the systematic introduction of actuator characteristics such as anti-saturation and rate-of-change limits, and improving the consistency and interpretability of simulation results with physical experiments, as shown in Table 1. The key points of modeling are as follows:

1. Vehicle body: the vehicle body is designed as a rigid body with a mass of 280 kg. A simplified envelope box model is used to improve computational efficiency.
2. Wheel system: each of the three wheels on both sides has an independent revolute joint with a wheel radius of 200 mm. The tires are modeled as a rigid simplified model, ignoring tread deformation effects.
3. Rocker-bogie mechanism: the rocker arms are modeled as passive revolute joints connected to the chassis and wheel modules. Joint constraints are used to preserve the mechanism geometry while allowing passive rotation under terrain contact and gravity. No active chassis pitch adjustment mechanism is assumed in the present simulation or prototype validation.
4. Terrain obstacles: a rigid step with a height of 250 mm and a 20° slope are constructed and connected to the ground as a fixed body.
5. Drive input: a closed-loop speed controller is constructed in Simulink. The ADAMS terminal outputs the angular velocity ωi(t) of each wheel axle. The angular velocity of each wheel axle revolute joint is used as feedback. After receiving the feedback, Simulink calculates the speed error e_i_(t) with the target angular velocity ω_ref_(t) (i.e., 1 rad/s). The expression is as follows:

(4) $$e_{i}\left(t\right)=\omega_{ref}\left(t\right)-\omega_{i}\left(t\right)$$

The torque command is calculated using a discrete PI structure.

(5) $$u_{i}\left(t\right)=K_{p}e_{i}\left(t\right)+K_{i}\int e_{i}\left(t\right)dt$$

**Figure 7:**
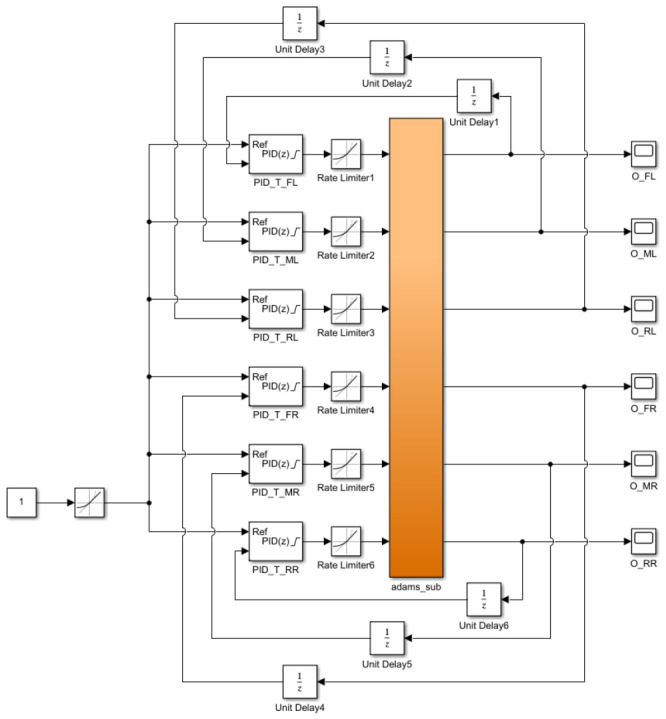
ADAMS-Simulink co-simulation block diagram for six-wheel independent drive speed closed-loop control.

To closely approximate the actual drive capability constraints, the control is set with a torque limit |u(t)| ≤ T_max_ =180 N·m, and a torque rate limiter is introduced to equivalently characterize the current loop bandwidth and torque build-up process, suppressing numerical spikes caused by obstacle-crossing contact transients. The generated u_i_(t) is written to the ADAMS system input channel through the Adams/Controls interface, and drive torque is applied to each wheel axle rotating joint, thus forming a closed loop, as shown in Figure 7.

#### 4.1.2. Definition of Simulation Scenarios

**Figure 8:**
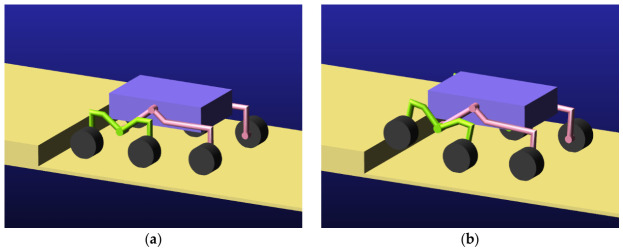

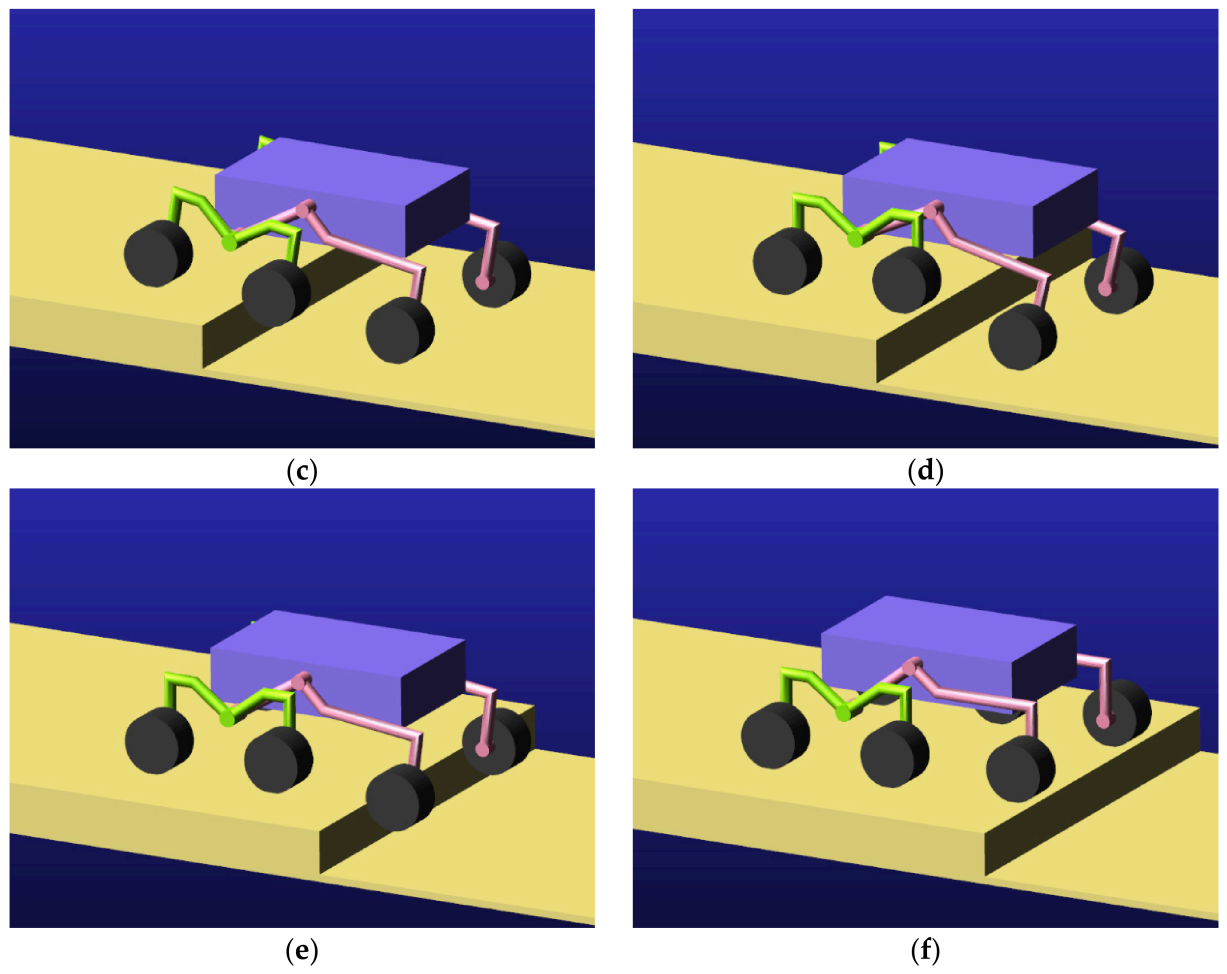
Six-wheel differential-steering mobile platform obstacle-crossing simulation: (**a**) preparing to cross obstacles; (**b**) front wheel obstacle-crossing; (**c**) middle wheel obstacle-crossing; (**d**) obstacle-crossing process; (**e**) rear wheel obstacle-crossing; (**f**) obstacle course completed.

**Figure 9:**
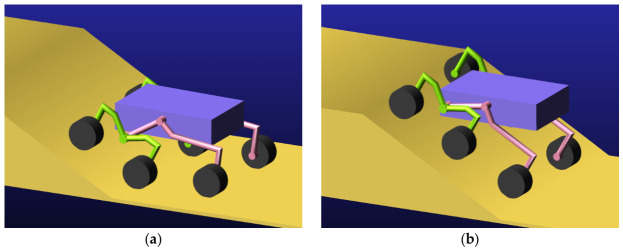

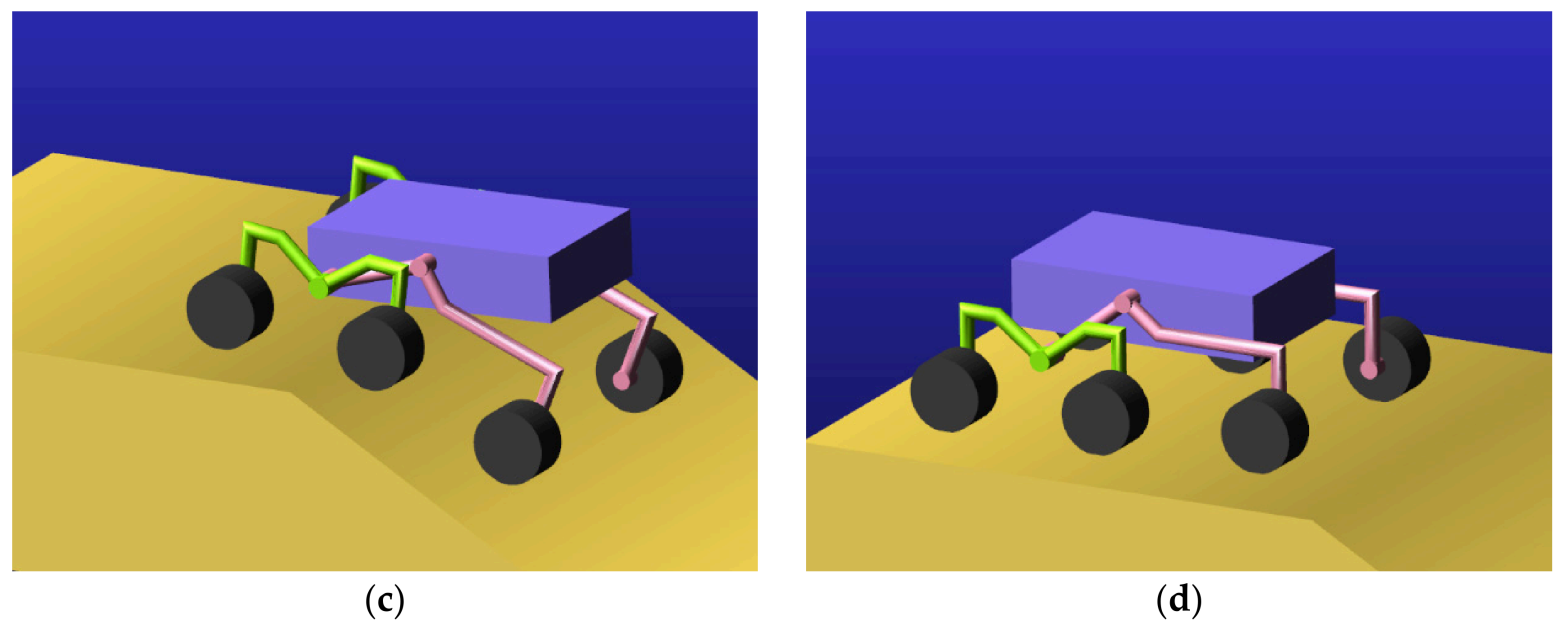
Simulation of a six-wheel differential-steering mobile platform climbing a 20° slope: (**a**) front wheel climbing; (**b**) climbing process; (**c**) the middle wheel climbed to the top of the slope; (**d**) climb completed.

To assess the robot’s trafficability within the scope of the present prototype validation, two representative operating scenarios were constructed:

1. Obstacle-crossing scenario: a rigid vertical step with a height of 250 mm (approximately 125% of the wheel radius) was set to evaluate whether the prototype could traverse a representative large step while keeping wheel torque within the actuator limit, as shown in Figure 8.

2. Slope climbing scenario: a slope with a 20° gradient was set to evaluate traction stability and drive-load margin under a continuous gravitational component, as shown in Figure 9.

### 4.2. Physical Prototype and Experimental Scenarios

Based on the mechanical design, a full-scale engineering prototype was developed. The prototype has a total mass of 280 kg and a wheel radius of 200 mm, and is powered by a 48 V lithium battery. The short-time allowable output torque of the motor-gearbox module is 180 N·m.

**Figure 10:**
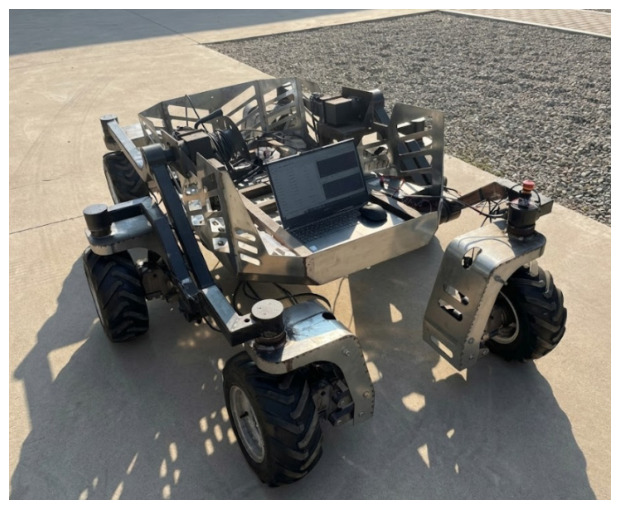
Flat ground walking and turning-in-place test.

Figure 10 shows the straight line driving and acceleration/deceleration tests. The platform was commanded by the upper computer to run at low speeds not exceeding 0.2 m/s. A stepped speed profile was used, with repeated tests at 0.05, 0.10, 0.15, and 0.20 m/s. The results showed that the platform started smoothly, with no obvious impact or vibration. During constant-speed motion, no significant trajectory deviation was observed. Laser measurements showed that the lateral offset over a 10 m straight path remained within ±10 mm, satisfying the design requirement. In the emergency braking test at 0.2 m/s, the average braking distance was 35 mm and the braking time was about 0.55 s. The vehicle posture remained stable during braking, with the pitch angle change below 5°, indicating a reasonable center-of-gravity design and effective suspension buffering.

The in-place steering test was conducted using a differential-drive strategy, in which the three left wheels rotated backward and the three right wheels rotated forward to achieve 360° pivot steering. Tests were performed at angular velocities of 0.2, 0.4, and 0.6 rad/s. The results showed that the platform could overcome tire–ground sliding resistance and complete in-place steering reliably, supported by the large wheel spacing and sufficient hub motor torque.

#### 4.2.1. Obstacle-Crossing Test Protocol

**Figure 11:**
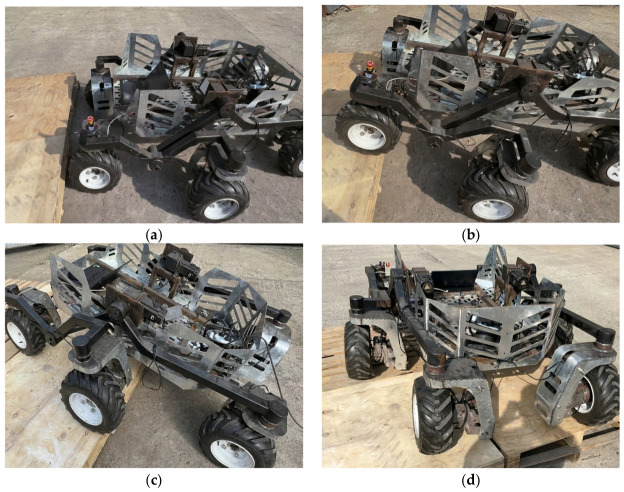
Obstacle course: (**a**) preparation stage; (**b**) front wheel obstacle-crossing; (**c**) middle wheel obstacle-crossing; (**d**) obstacle course completed.

The experiment used a 250 mm wooden step to represent a controlled geometric obstacle and to provide a repeatable mechanical mobility test. The test was not intended to reproduce radiation, contamination, dust, or other nuclear environment exposure conditions, as shown in Figure 11.

1. Procedure: the robot approached and traversed the step at a low speed of 0.2 m/s. The test recorded the full process from front wheel contact to completion of rear wheel crossing.
2. Objective: capture the instantaneous torque response during the three critical stages—front wheel contact, middle wheel lifting, and rear wheel crossing—to evaluate whether the passive rocker-bogie chassis and drive system could complete the specified step traversal without prolonged overload.

#### 4.2.2. Slope Climbing Test Protocol

**Figure 12:**
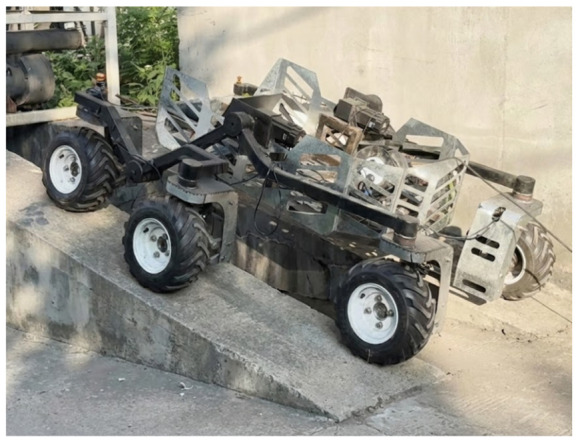
Hill climbing test.

A 20° concrete slope was used as a controlled inclined surface for short-duration mobility testing. This test evaluates slope climbing feasibility and drive-load margin under ordinary outdoor conditions rather than environmental hardening for nuclear deployment, as shown in Figure 12.

1. Procedure: the robot started from flat ground, entered the slope at a constant set speed of 0.2 m/s, and maintained a short steady climbing state after all wheels were on the slope.
2. Objective: monitor the current-derived torque of the six wheels during the transition and short steady-state climbing phase to evaluate drive-load margin and possible traction interruption on the 20° slope.

## 5. Results and Discussion

### 5.1. Obstacle-Crossing Performance

**Figure 13:**
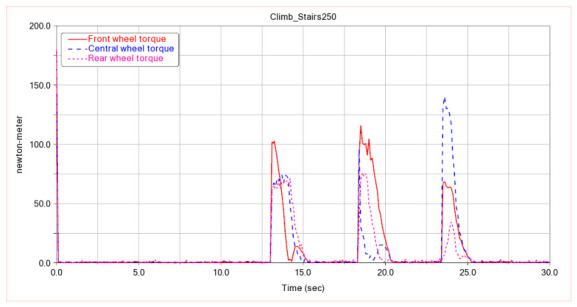
Obstacle-crossing simulation driving torque curve.

**Figure 14:**
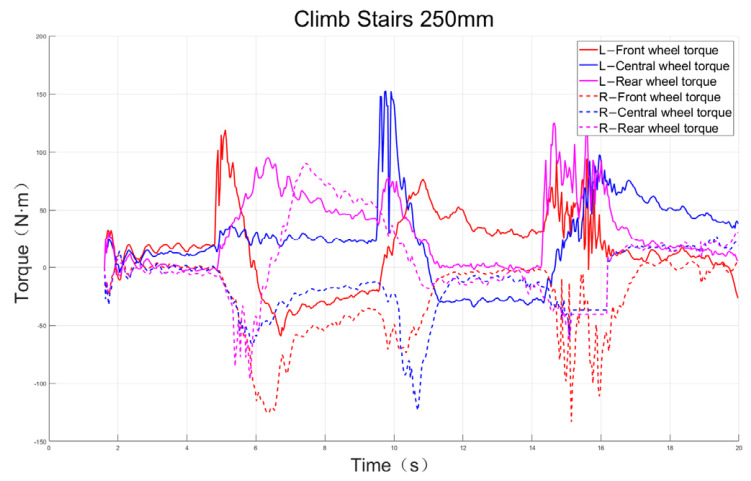
Experimental wheel-drive torque curve during 250 mm step traversal.

To assess the drive-load response under real-world obstacle-crossing conditions, ADAMS/Simulink simulation data and prototype experimental data were compared. Figure 13 and Figure 14 illustrate the time-domain response of the driving torque when the robot traverses a 250 mm step.

#### 5.1.1. Simulation Result Analysis

Figure 13 illustrates the torque output curves of the driving wheels under ideal simulation conditions in ADAMS.

It should be noted that this simulation is based on an ideal symmetric model, assuming the robot faces the obstacle squarely and the forces on the left and right sides are identical. Therefore, the figure only displays the torque variations in the three wheels on a single side (the left side). Two significant dynamic characteristics can be observed from the curves:

1. Clear timing sequence: the torque peaks appear strictly in the order of the front, middle, and rear wheels, corresponding to the climbing moments around t = 13 s, 18 s, and 23 s, respectively.
2. All-positive drive: due to the ideal symmetry assumption, all wheels output positive driving torque to overcome gravity. The peak torque is approximately 100–140 N·m, and the curves are smooth with no negative values.

As shown in Table 2, the simulated torque response during the step traversal process is summarized as follows.

1. Front wheel: it exhibits a large initial peak torque, playing a critical role in the initial climbing stage. The sudden surge indicates a strong load response upon its first contact with the step.
2. Central wheel: it bears the maximum torque, serving as the key support wheel. Particularly in the latter stage of obstacle-crossing, the central wheel sustains the heaviest load, suggesting that the structural design or the center of mass (CoM) may be biased towards the center.
3. Rear wheel: its participation shows a time lag, with relatively smaller and stable torque. This indicates that its structural role is likely focused on auxiliary propulsion or maintaining postural stability.

#### 5.1.2. Analysis of Experimental Results

Figure 14 records the real-time torque data for all six wheels of the prototype while traversing the same obstacle in a real physical environment (calculated based on current feedback).

1. Sign convention of drive data: unlike the simulation graph, which displays the torque magnitude for one side, the experimental data present positive values on one side and negative values on the other side.

Left wheels (solid lines): torque is positive (T > 0).

Right wheels (dashed lines): torque is negative (T < 0).

This sign difference is determined by the topological installation and coordinate definition of the drive system: the left and right motors are mounted in a mirrored configuration. To achieve forward movement of the vehicle, the left motors rotate in the positive direction, while the right motors rotate in the negative direction. Therefore, the negative values for the right wheels represent effective driving torque, not braking. This observation is a coordinate/sign convention issue and should not be interpreted as a unique dynamic torque-distribution mechanism.

2. Transient shock in real-world obstacle-crossing: the experimental data exhibit intense pulse-like fluctuations. Particularly for the front (red curves) and central (blue curves) wheels, their torque magnitude (absolute value) climbs rapidly to around 150 N·m at the moment of impact. The measured torque fluctuations should be interpreted primarily as transient impact loads and measurement-chain fluctuations during wheel–step engagement. Because the physical obstacle was a concrete step and the wheels used pneumatic tires, the sustained high-frequency components cannot be attributed simply to hard contact with a concrete step.

#### 5.1.3. Comparative Discussion

By comparing the simulation (Figure 13) and experimental (Figure 14) data, the dynamic characteristics of the drive system can be discussed from three aspects: timing sequence, sign convention, and magnitude discrepancy.

1. Consistency in obstacle-crossing sequence and trend: despite numerical differences, the experimental results show the same qualitative order of wheel loading as the simulation. The torque peaks of the front, central, and rear wheels appear sequentially, corresponding to the geometric process of step traversal. This supports the use of the multibody simulation as a qualitative design tool for identifying the order and approximate magnitude of drive-load demand.
2. Mechanism of the positive–negative torque signs: the most visible difference between simulation and experiment is the positive/negative sign distribution of the recorded torques. This phenomenon stems from the mirrored installation of the left and right drive units and the adopted coordinate definition. It represents neither dynamic antagonism nor a special load-sharing mechanism. Accordingly, the following analysis uses torque magnitude when comparing the left and right sides.
3. Transient impact and magnitude discrepancy: the instantaneous torque peaks measured in experiments (approx. 150 N·m) are notably higher than the simulation predictions (approx. 120 N·m), accompanied by intense high-frequency oscillations. The primary reasons for this discrepancy include:

Hard contact effect: ADAMS simulations typically use the penetration depth method to calculate contact forces, which introduces a certain numerical smoothing effect. In contrast, the physical prototype involves direct impact between pneumatic off-road wheel–tire assemblies and concrete steps, generating significant instantaneous impact loads.

Non-ideal factors: the experimental data capture additional resistance from gearbox backlash, bearing friction, and terrain unevenness, which were simplified in the ideal model.

Asymmetric loading: the observed magnitude asymmetry between left and right wheels in the experiment (e.g., the right front wheel exerting more force than the left at certain moments) reflects the difficulty of maintaining perfect heading alignment in real-world obstacle-crossing. This uneven loading results in one side bearing a larger instantaneous load.

Although the real environment introduces shock loads and asymmetry, the sensor data show that the peak torques of the drive motors during obstacle-crossing remained within their short-time allowable range. The results indicate that, under the tested open-loop operating condition, the passive rocker-bogie chassis and drive system completed the specified 250 mm step traversal.

As shown in Table 3, the overall trend of the simulation results and the test results is basically consistent under the obstacle-crossing conditions. However, there are significant deviations in the local peaks. Specifically, the peak error of the rear wheel during the front wheel obstacle-crossing stage is as high as 150%, and the peak error of the rear wheel during the rear wheel obstacle-crossing stage reaches 66.7%. The analysis suggests that the errors mainly result from factors such as nonlinear deformation of the tires, random changes in the ground friction coefficient, and mechanical transmission clearance.

### 5.2. Slope Climbing Performance

**Figure 15:**
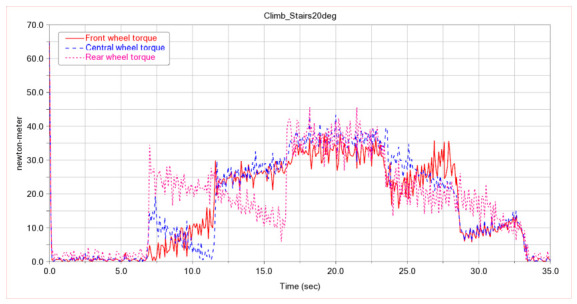
Simulation driving torque curve for hill climbing.

**Figure 16:**
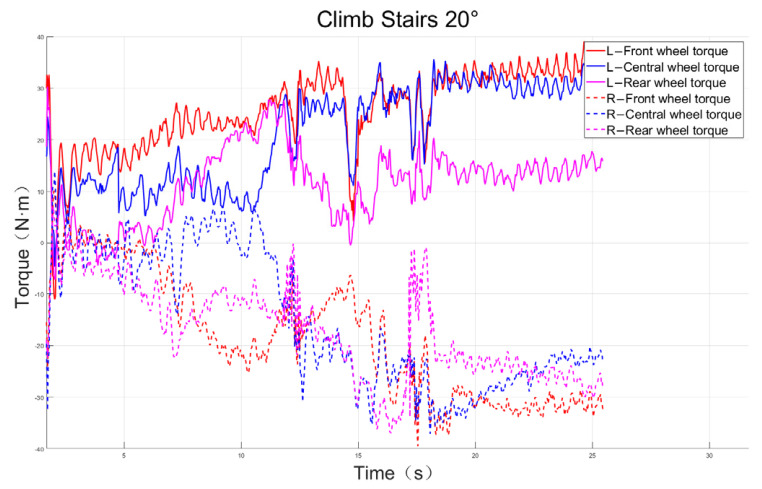
Experimental wheel-drive torque curve during 20° slope climbing.

To verify the robot’s continuous load-bearing capacity on long ramps, we conducted climbing tests on a 20° concrete slope. Figure 15 and Figure 16 record the variations in driving torque under simulation and experimental environments, respectively.

#### 5.2.1. Simulation Result Analysis

Figure 15 illustrates the torque curves of the single-side wheels as the robot climbs a 20° slope in the ADAMS simulation.

1. Sequential load entry: the simulation curves reflect the process of the robot entering the slope. Around t = 7 s, the front wheel enters the slope first and its torque rises to overcome the gravitational component. Around t = 12 s, the middle wheel and rear wheel successively enter the slope, causing their torques to rise accordingly.
2. Steady-state load characteristics: when the robot is fully positioned on the slope (t = 17–28 s), the torque of all wheels enters a relatively stable plateau phase, maintaining a range of approximately 30–40 N·m. This represents the theoretical equilibrium torque required for constant-speed climbing under the simulated condition.

#### 5.2.2. Analysis of Experimental Results

Figure 16 records the torque response of all six wheels of the prototype during the actual slope climbing process.

It should be noted that, due to the limited length of the ramp at the experimental facility, this test primarily recorded the process of the robot transitioning from flat ground to the slope and establishing a short steady climbing state; it does not include long-duration endurance data. Therefore, the results are used to evaluate short-duration climbing feasibility and drive-load margin on a 20° slope, rather than long-term thermal endurance.

1. Sign convention of drive data: unlike the simulation, which shows single-side torque magnitude, the experimental data present positive values on the left side and negative values on the right side. This is caused by mirrored motor installation and the adopted coordinate definition. The negative values for the right wheels represent effective forward driving torque, not braking; therefore, torque magnitude is used for load comparison.
2. Process analysis: the data show that, around t = 12 s, as all six wheels fully entered the slope surface, the torque of each wheel quickly converged into a steady-state range, maintaining between 25 and 35 N·m (absolute value).
3. Stability observation: although the data duration is limited, the curves did not show severe oscillations, divergence, or complete torque loss during this period. The result suggests no obvious traction interruption under the tested condition; however, direct wheel-load measurement is required for confirmation.

As shown in Table 4, the overall trend of the simulation results is basically consistent with that of the experimental results, but there are significant deviations in some local peaks. Specifically, the peak error of the rear wheel during the climbing stage reaches 200%, and the peak error of the front wheel during the climbing stage reaches 600%. The analysis suggests that the errors mainly result from factors such as the nonlinear deformation of the tires, the random variation in the ground friction coefficient, and the mechanical transmission gap.

#### 5.2.3. Comparative Discussion

1. Numerical comparison: the simulation predicted an average climbing torque of approximately 35 N·m, while the experimentally measured steady-state torque magnitude was approximately 30–32 N·m. The experimental values are slightly lower than the simulation predictions (approximately 10% deviation). This level of agreement is considered sufficient for preliminary drive-load estimation, but it should not be interpreted as a complete validation of an analytical closed-form dynamic model.
2. Motor safety verification: experimental data indicate that, during the short steady climbing phase, the average load per wheel was approximately 30 N·m. This value is below the rated torque (40 N·m) and well within the short-time allowable range of the motor-gearbox module, indicating an adequate drive-load margin for the tested 20° slope condition.
3. Configuration assessment: no complete torque drop to zero was observed in the experimental curves during the tested slope climbing interval. This suggests that no obvious wheel lift-off occurred under the tested condition. However, because no wheel load cells or direct normal-force sensors were installed, this result is reported as an indirect observation rather than direct proof of equal weight distribution among all six wheels.

## 6. Conclusions and Future Work

### 6.1. Conclusions

Addressing the needs for attachment replacement and environmental adaptability in nuclear decommissioning tasks, this paper designed and implemented a six-wheel mobile platform with a passive rocker-bogie suspension and six independent drive units. Prototype tests over a 250 mm step and on a 20° slope were conducted. The main conclusions are as follows:

1. Configuration and implementation: the prototype adopts a well-known passive rocker-bogie suspension architecture combined with six-wheel independent drive. The contribution of this work lies in adapting and experimentally evaluating this configuration for a heavy-duty nuclear environment support platform, rather than proposing a new suspension topology or an active chassis pitch adjustment mechanism.
2. Verification under representative terrain conditions: prototype experiments demonstrated that the platform successfully surmounted a 250 mm vertical obstacle and climbed a 20° slope under the tested conditions. These tests verify the feasibility of the prototype for the specified representative terrains, but they are not sufficient to claim generalized obstacle-crossing capability beyond the tested cases.
3. Drive-load evaluation via onboard sensor data: real-time monitoring data from the six-wheel drives showed that the motor torques remained within the allowable range during the tested obstacle-crossing and slope climbing processes. The measured positive/negative torque signs are explained by mirrored motor installation. Because vertical wheel loads were not directly measured, claims regarding equal load distribution among all wheels have been removed and are left for future validation using wheel load cells or contact-force instrumentation.

### 6.2. Future Work

To further enhance the operational capability and adaptability of the platform in real-world nuclear environments, building upon the mechanical and dynamic foundations validated in this paper, future work will focus on the following directions:

1. Multi-modal perception, wheel-load measurement, and closed-loop control: building on the current proprioceptive feedback (current and encoders), future work will integrate high-precision 3D vision (LiDAR/depth cameras), force/tactile sensors, and wheel-load measurement. These additions will support direct validation of wheel-normal-force distribution and closed-loop terrain-adaptive control.
2. Optimization of obstacle-crossing strategies: we aim to construct an obstacle-crossing action primitive library based on dynamic constraints. By introducing reinforcement learning or terrain-adaptive algorithms, the robot will be able to intelligently select the optimal approach trajectory, wheel speed commands, and torque-control strategies according to real-time terrain features (e.g., step height, slope friction).
3. System integration for attachment replacement: The six-wheel differential-steering chassis will be integrated physically and logically with a multi-degree-of-freedom manipulator system. Experiments on “Eye–Hand–Leg” coordination will be conducted under obstacle-crossing or tilted terrain conditions, focusing on validating automated precise docking technologies based on visual servoing and force control.
4. Nuclear environment adaptability assessment: targeting high-radiation operational scenarios, radiation hardening tests will be performed on key electronic components and sensors. The long-term stability and reliability of the entire system in radiation environments will be evaluated to provide engineering data support for final deployment in decommissioning projects.

## Figures and Tables

**Table 1:** Simulation parameters.

Serial Number	Settings	Central Wheel (N·m)
1	Vehicle body weight	280 kg
2	Wheel diameter	0.2 m
3	Wheel speed	0.2 m/s
4	Stiffness	1.0 × 10^8^
5	Damping	1.0 × 10^4^
6	Penetration depth	1.0 × 10^−4^
7	Friction	0.8

**Table 2:** Simulated wheel-drive torque during 250 mm step traversal.

Time (s)	Front Wheel (N·m)	Central Wheel (N·m)	Rear Wheel (N·m)
0–13	<5	~5	~5
13–14	~100	~75	~70
15	~14	--	--
18–20	~130	~96	~75
22	--	~15	--
23–26	~60	~140	~35
30	--	--	~7

**Table 3:** Overcoming obstacles quantitative comparison.

Obstacle-Crossing Wheel Position	Rung Position	Peak Error (%)	Source of Relative Error
Middle wheel obstacle-crossing	Front wheel	8.33	The tires have slightly deformed
	Middle wheel	150	Mechanical clearance + friction changes
	Rear wheel	22.2	Tire deformation + friction changes
Middle wheel obstacle-crossing	Front wheel	53.3	Mechanical clearance
	Middle wheel	40.0	Friction changes
	Rear wheel	0	Almost no error
Rear wheel obstacle-crossing	Front wheel	38.0	Tire deformation + mechanical clearance
	Middle wheel	30.0	Friction changes
	Rear wheel	66.7	Tire deformation

**Table 4:** Gradient-based quantitative comparison.

Obstacle-Crossing Wheel Position	Rung Position	Peak Error (%)	Source of Relative Error
Middle wheel obstacle-crossing	Front wheel	75	Slight deformation of the tire
	Middle wheel	66.7	Mechanical clearance + friction variation
	Rear wheel	600.0	Tire deformation + friction variation
Middle wheel obstacle-crossing	Front wheel	0	Almost no error
	Middle wheel	20.0	Friction variation
	Rear wheel	30.0	Friction variation + mechanical clearance
Rear wheel obstacle-crossing	Front wheel	16.7	Tire deformation + mechanical clearance
	Middle wheel	9.1	Friction variation
	Rear wheel	200	Tire deformation + friction variation

## Data Availability

All data included in this study are available upon request by contact with the corresponding author.
